# Altered offspring neurodevelopment in an L-NAME-induced preeclampsia rat model

**DOI:** 10.3389/fped.2023.1168173

**Published:** 2023-07-13

**Authors:** Noriyuki Nakamura, Takafumi Ushida, Atsuto Onoda, Kazuto Ueda, Ryosuke Miura, Toshihiko Suzuki, Satoru Katsuki, Hidesuke Mizutani, Kosuke Yoshida, Sho Tano, Yukako Iitani, Kenji Imai, Masahiro Hayakawa, Hiroaki Kajiyama, Yoshiaki Sato, Tomomi Kotani

**Affiliations:** ^1^ Department of Obstetrics and Gynecology, Nagoya University Graduate School of Medicine, Nagoya, Japan; ^2^ Department of Obstetrics and Gynecology, Anjo Kosei Hospital, Aichi, Japan; ^3^ Division of Reproduction and Perinatology, Center for Maternal-Neonatal Care, Nagoya University Hospital, Nagoya, Japan; ^4^ Faculty of Pharmaceutical Sciences, Sanyo-Onoda City University, Yamaguchi, Japan; ^5^ Division of Neonatology, Center for Maternal-Neonatal Care, Nagoya University Hospital, Nagoya, Japan

**Keywords:** preeclampsia, neurodevelopment, L-NAME, proteomics, cerebrospinal fluid

## Abstract

**Introduction:**

To investigate the mechanism underlying the increased risk of subsequent neurodevelopmental disorders in children born to mothers with preeclampsia, we evaluated the neurodevelopment of offspring of a preeclampsia rat model induced by the administration of N-nitro-L-arginine methyl ester (L-NAME) and identified unique protein signatures in the offspring cerebrospinal fluid.

**Methods:**

Pregnant rats received an intraperitoneal injection of L-NAME (250 mg/kg/day) during gestational days 15–20 to establish a preeclampsia model. Behavioral experiments (negative geotaxis, open-field, rotarod treadmill, and active avoidance tests), immunohistochemistry [anti-neuronal nuclei (NeuN) staining in the hippocampal dentate gyrus and cerebral cortex on postnatal day 70], and proteome analysis of the cerebrospinal fluid on postnatal day 5 were performed on male offspring.

**Results:**

Offspring of the preeclampsia dam exhibited increased growth restriction at birth (52.5%), but showed postnatal catch-up growth on postnatal day 14. Several behavioral abnormalities including motor development and vestibular function (negative geotaxis test: *p* < 0.01) in the neonatal period; motor coordination and learning skills (rotarod treadmill test: *p* = 0.01); and memory skills (active avoidance test: *p* < 0.01) in the juvenile period were observed. NeuN-positive cells in preeclampsia rats were significantly reduced in both the hippocampal dentate gyrus and cerebral cortex (*p* < 0.01, *p* < 0.01, respectively). Among the 1270 proteins in the cerebrospinal fluid identified using liquid chromatography-tandem mass spectrometry, 32 were differentially expressed. Principal component analysis showed that most cerebrospinal fluid samples achieved clear separation between preeclampsia and control rats. Pathway analysis revealed that differentially expressed proteins were associated with endoplasmic reticulum translocation, Rab proteins, and ribosomal proteins, which are involved in various nervous system disorders including autism spectrum disorders, schizophrenia, and Alzheimer's disease.

**Conclusion:**

The offspring of the L-NAME-induced preeclampsia model rats exhibited key features of neurodevelopmental abnormalities on behavioral and pathological examinations similar to humans. We found altered cerebrospinal fluid protein profiling in this preeclampsia rat, and the unique protein signatures related to endoplasmic reticulum translocation, Rab proteins, and ribosomal proteins may be associated with subsequent adverse neurodevelopment in the offspring.

## 1. Introduction

Preeclampsia (PE) affects 2%–8% of all pregnancies and is a life-threatening complication that is accompanied by multi-organ dysfunction. Uteroplacental mismatch attributed to abnormal trophoblast invasion in early pregnancy is central to the pathogenesis of PE (1). Placental syncytiotrophoblast stress-derived factors and imbalance between pro- and anti-angiogenic factors contribute to systemic endothelial dysfunction, followed by clinical manifestations of new-onset hypertension and various multi-organ damage (e.g., proteinuria, eclampsia, HELLP syndrome, liver dysfunction, coagulation abnormalities, and posterior reversible encephalopathy syndrome) (1, 2).

Long-term population-based cohort studies and meta-analyses have accumulated evidence of various neurological and neurodevelopmental disorders [e.g., cerebral palsy, periventricular leukomalacia, intraventricular hemorrhage, autism spectrum disorder (ASD), attention-deficit/hyperactivity disorder, intellectual disability, cognitive disability, and anxiety] in offspring born to mothers with PE (3–5). To date, many researchers have investigated the mechanisms underlying adverse neurodevelopment in offspring attributed to intrauterine exposure to PE using various animal models of PE (3). Previous animal studies have demonstrated various alterations in (1) neuroanatomy (e.g., reduced volumes of cerebral neocortex, caudate–putamen, occipital lobe, and entorhinal cortex) and vascularization (e.g., small diameter vessels) in the central nervous system (6, 7); (2) the number and localization of immune cells (e.g., microglia), apoptotic cells, and inflammatory cytokines (8, 9); (3) neurogenesis, gliosis, and myelination (10); and (4) protein expression both in quality and quantity by western blotting and brain tissue pathology in the offspring of PE model animals (3). However, the pathogenesis of the increased risk of such disorders in offspring remains unclear (7, 11). In this study, we sought to evaluate whether altered neurodevelopment in offspring is observed in a PE rat model induced by N-nitro-L-arginine methyl ester (L-NAME: a nonselective inhibitor of nitric oxide synthase) and is consistent with clinical consequences in humans. The L-NAME-induced PE rat model is recognized as a typical PE model and is widely used for PE research (12).

In addition, alterations in cerebrospinal fluid (CSF) profiling in offspring using an animal model of PE have not been described in the literature. In general, CSF is not readily accessible compared with other biofluids, including blood, saliva, and urine, in clinical and experimental settings (13). However, CSF is the most proximal to the central nervous system and includes brain-derived components; therefore, it accurately reflects the disease-related pathology of various neurological disorders and may represent a promising matrix for the identification of clinical biomarkers and unique protein profiling by proteome analysis (14–16).

Thus, we aimed to evaluate the neurodevelopment of the offspring of the L-NAME-induced PE rat model and identify unique protein signatures in the CSF of the offspring on postnatal day (PND) 5. Although the technique of collecting CSF at this early stage of life is challenging, protein profiling on PND 5 should directly reflect the effects of exposure to maternal PE without the effects of various factors associated with the postnatal environment. This study may provide potential biomarkers of neurological disorders in offspring as well as insights into the biological pathways and mechanisms associated with such disorders.

## 2. Methods

### 2.1. Animal model

**Figure 1:**
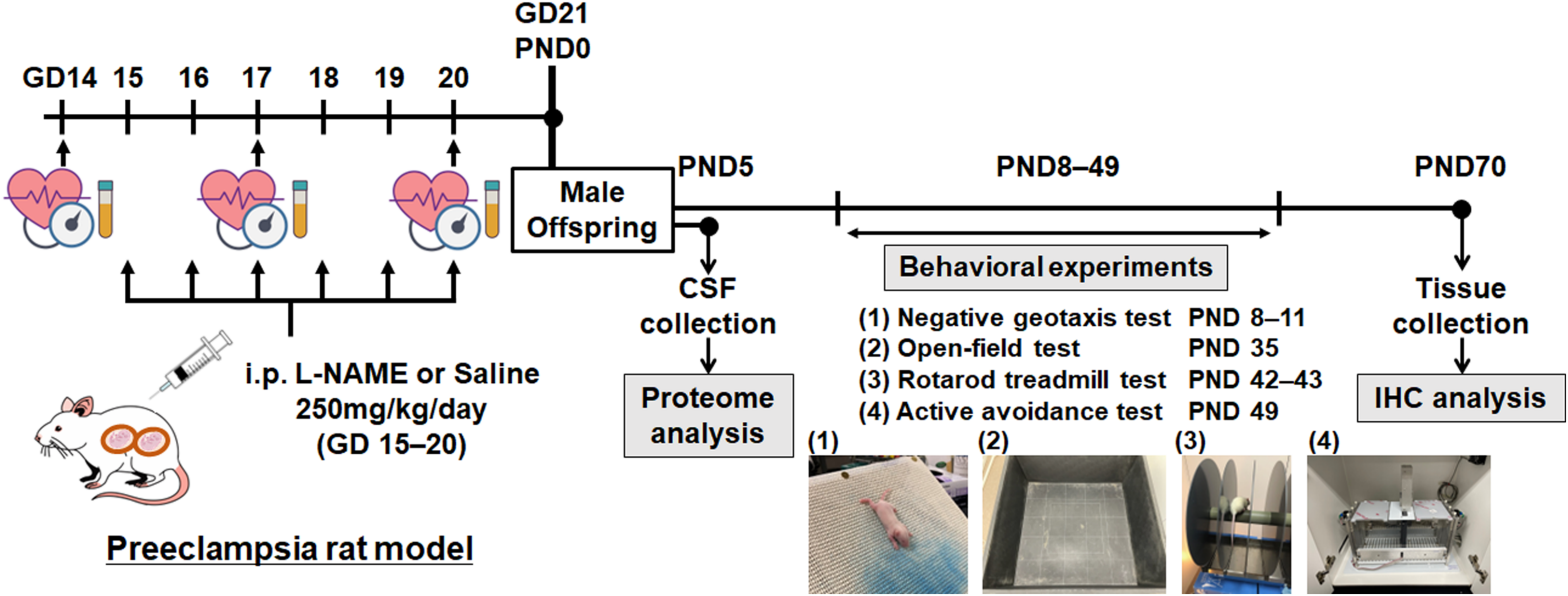
Overview of the experimental protocol. Schematic representation of the experimental protocol. GD, gestational day; PND, postnatal day; CSF, cerebrospinal fluid; IHC, immunohistochemistry.

Pregnant Sprague-Dawley rats at gestational day (GD) 13 (8–12 weeks of age) were purchased from SLC Laboratory Animal Co. Ltd. (Shizuoka, Japan) and bred at the Laboratory Animal Care Center of Nagoya University. The rats were individually housed under controlled temperature (25 °C) in a 12 h light/dark cycle with *ad libitum* access to food and water. Rats were randomly assigned to the PE (*n* = 6) and control (*n* = 7) groups. L-NAME was purchased from Sigma-Aldrich (N5751-10G, Tokyo, Japan). Pregnant rats in the PE group received an intraperitoneal (i.p.) injection of L-NAME (250 mg/kg/day) from gestational days 15 to 20. Pregnant rats in the control group received an i.p. injection of saline (250 mg/kg/day) during the same period. Pregnant rats were allowed to deliver normally, and pups were culled to eight (four males and four females per litter, if possible) on PND 5 to minimize the impact of litter size on subsequent experimental outcomes. In this study, pups were fostered by their dams that received i.p. injections of L-NAME or saline. Consequently, daily i.p. injections to dams could induce additional stress. However, the stress was uniformly distributed among the two groups, as both groups received an equal number of i.p. injections. Male offspring were placed in separate cages at the time of weaning (PND 21), and all subsequent experiments were performed on male offspring only. Four behavioral experiments were performed on PND 8–49. All rats were transcardially fixed with 4% paraformaldehyde on PND 70, and brain tissue samples were collected and stored at −30 °C in a cryoprotectant solution. Figure 1 shows the experimental protocol used in this study. All animal experiments were performed in compliance with the guidelines for animal care and use of laboratory animals, and the experimental protocol was approved by the Institutional Animal Care Committee (approval number: M210424).

### 2.2. Measurement of blood pressure and urinary protein level

Blood pressure during pregnancy was non-invasively measured on GD 14, 17, and 20 using an automatic blood pressure analyzer with a tail-cuff device (BP-98AL; Softron, Tokyo, Japan) after the rats received pre-training for blood pressure measurements once on GD 13 to acclimate the rats to the experimental equipment. The mean value of five readings for each rat was recorded after the rats were placed in a heating chamber maintained at 37 °C for approximately 5–10 min. Urinary samples were collected on GDs 14, 17, and 20, and urinary protein levels (protein/Cre) were measured using the Pierce BCA Protein Assay Kit (Thermo Fisher Scientific, Tokyo, Japan) and Creatinine ELISA kit (Exocell, Newtown Square, Pennsylvania) according to the manufacturer's instructions.

### 2.3. Behavioral experiments

All behavioral experiments were performed by the same researcher who performed i.p. injections to the dams. However, the rats were assigned consecutive numbers so that the group assignment was unknown to the researcher. We performed four behavioral experiments to assess the neonatal reflex (negative geotaxis test) and three behavioral analyses of juvenile rats (open-field test, rotarod treadmill test, and active avoidance test). The experimental protocol is described in detail below.

#### 2.3.1. Negative geotaxis test

Negative geotaxis is an innate postural response and a directional movement against gravity. The negative geotaxis test is an early behavioral test; it was performed on PND 8–11 in this study to evaluate motor development and vestibular function and is commonly used in the study of brain injury, central nervous system diseases, and neurodevelopmental disorders (17, 18). Pups were individually placed head-down on a sloped platform (30°), and the latency to complete the geotaxis response was recorded. The results of the negative geotaxis test were scored from 0 to 5 according to latency, as previously described: 0 = no response during 60 s, 1 = ≥60 s or falling down a slope, 2 = 45–60 s to complete the geotaxis response, 3 = 30–45 s, 4 = 15–30 s, and 5 = <15 s (17). This experiment presents a potential risk of bias because a researcher could infer the group allocation if there is a significant difference in body weight between the two groups during the experimental period.

#### 2.3.2. Open-field test

The open-field test is widely used to assess locomotor activity, anxiety-like behavior, and exploratory behavior in rodents (19). The animal was placed in the middle of a square apparatus (100 × 100 cm) with a grid of 20 × 20 cm squares on its base, and its movements were recorded using a video camera for 5 min (17). The following parameters were evaluated using an automated tracking system (ANY-maze Video Tracking System; Stoelting Co., Wood Dale, IL, USA): (1) total distance moved (m), (2) average speed (m/s), (3) time immobile (s), and (4) entries in the central area (n). The open-field test was performed on PND 35 (20).

#### 2.3.3. Rotarod treadmill test

Rotarod treadmill test was performed to assess balance and motor coordination in rodents on PND 42–43 (17, 21). Each rat was placed on a rotating rod (47750; Ugo Basile, Gemonio, Italy), which gradually accelerated the rotation speed from 5 to 40 rpm over 5 min. Two consecutive experiments were performed for each rat, and the time spent on the rotating rod between placement on the rod and falling off was recorded and averaged (maximum 300 s).

#### 2.3.4. Active avoidance test

An active avoidance test was performed to evaluate fear-motivated associative learning and memory, as previously described (17, 22). The test was conducted on PND 49 in an automated shuttle box (Med Associates Inc., St. Albans, Vt., USA). The test consisted of 10 trials of conditioned stimulus (light and buzzer tone) and aversive unconditioned stimulus (foot shock). The average interval time between each trial was 30 s, ranging from 10 to 90 s. Over repeated trials, the rats learned to avoid an aversive stimulus by changing the shuttle box. The parameters were analyzed using the MED-PC IV program (Med Associates Inc.). Learning performance was evaluated using the avoidance rate in six consecutive sections.

### 2.4. Immunohistochemistry

After the completion of all behavioral experiments, the rats were anesthetized with 5% isoflurane and i.p. somnopentyl (0.5 ml) on PND 70 and transcardially perfused with 4% paraformaldehyde. Brain tissues were collected and fixed with 4% paraformaldehyde, followed by 30% sucrose for cryoprotection. Tissues were cut into 50-μm sections using a microtome throughout the brain every 600 mm. To evaluate the hippocampal dentate gyrus and cerebral cortex, coronal sections of the brain were examined (bregma −1.4 mm to −8.6 mm). The sections were blocked with 0.6% H_2_O_2_ in phosphate buffered saline (PBS), then blocked using 3% normal donkey serum with 0.1% Triton-X100 in PBS for 30 min. Free-floating sections were stained with anti-neuronal nuclei (NeuN; product No. MAB377; dilution 1: 400; Merck Millipore) and secondary antibody (1:1,000; biotinylated donkey anti-mouse; Jackson ImmunoResearch Laboratories) (21). To evaluate the whole hippocampal dentate gyrus and cortex, the average numbers of tissue sections per brain were 8 and 12, respectively. Cell counts for positive cells per unit volume (mm^3^) were quantified using unbiased stereological counting techniques as previously described (Stereo Investigator version 10 stereology software, Micro Bright Field Europe EK, Magdeburg, Germany) (21).

### 2.5. Proteome analysis

CSF samples (PE [*n* = 10] and control [*n* = 10]) were obtained on PND 5 as previously described (23, 24). CSF samples were collected using transcutaneous cisterna magna puncture method with a microcap with outer and inner diameters of 0.90 mm and 0.63 mm, respectively (20 μl, Drummond Scientific Company, Broomall, PA, USA). After centrifugation, supernatants were stored at −80 °C. Each sample was diluted with phosphate-buffered saline to 0.5 μg protein/μl. Mass spectrometric analysis was performed as previously described (25). Briefly, proteins from CSF samples were processed for trypsin digestion for 16 h at 37 °C after reduction reactions and alkylation. Liquid chromatography-tandem mass spectrometry (LC-MS/MS) was performed to analyze the peptides on an UltiMate3000 RSLCnano LC system (Dionex Co., Amsterdam, Netherlands) in combination with an Orbitrap Fusion mass spectrometer (Thermo Fisher Scientific Inc., Waltham, MA, USA). Reversed-phase chromatography was performed with a linear gradient (0 min, 5% B; 100 min, 40% B) of solvent A (2% acetonitrile with 0.1% formic acid) and solvent B (95% acetonitrile with 0.1% formic acid). Peptide digests were loaded onto a nano HPLC capillary column (15 cm length, 75 μm i.d.) (Nikkyo Technos Co., Japan). Peptides were eluted at an estimated flow rate of 300 nl/min. Then, they were injected into a nanoelectrospray ion source for ionization.

A precursor ion scan was performed within a selected window of 400–1,600 mass to charge ratio (m/z) prior to MS/MS analysis. Tandem MS was performed by isolation at 0.8 Th with a quadrupole for high-energy collision dissociation fragmentation. The normalized collision energy was 30%. Tandem mass spectra were analyzed using an ion trap at a rapid scan rate. Only precursors with charge states of 2–6 were sampled for MS/MS. The dynamic exclusion duration was set to 15 s with 10 ppm tolerance.

Raw data were processed using Proteome Discoverer 1.4 (Thermo Fisher Scientific). A database search for protein identification was performed using the Mascot search engine version 2.6.0 (Matrix Science Inc., Boston, MA, USA). Protein identifiers (UniProt-KB accession numbers) and the corresponding expression values of the two groups for each identified protein were obtained from the dataset after filtering the rat database.

The data were converted to relative values, and proteins present in <4/10 samples in each group were excluded from further analysis. The data were then processed by quantile normalization using the preprocessCore package (ver. 1.58.0) in the R software (ver. 4.2.2). The relative protein values of the two groups were compared using Welch's *t*-test, and the False Discovery Rate (Benjamini-Hochberg method) was calculated. While there is no universally established definition for differentially expressed proteins in proteome analysis, we defined differentially expressed proteins between the two groups in this study based on the criteria of |log_2_ fold change (PE/control)| >0.8 and *q*-value <0.05. The cut-off value was determined based on the number of differentially expressed proteins for subsequent enrichment analysis. A heat map of protein expression profiles was created using Heatmapper (http://www.heatmapper.ca/) after convertion to z-scores. The volcano plot illustrates differentially abundant proteins. The logarithm to base 2 of the fold change (PE/control) is plotted on the X-axis, and the negative logarithm to base 10 of the *q*-value is plotted on the Y-axis. The vertical lines indicate log_2_ fold change = ± 0.8, and the horizontal line denotes *q*-value = 0.05. Enrichment analysis for Gene Ontology (GO) terms was performed using Metascape (http://metascape.org) after exclusion of unmapped differentially expressed proteins after filtering the rat database. Principal component analysis (PCA) was performed to visualize the distribution and combination of all samples using the rgl package (ver. 0.110.2) (26).

### 2.6. Statistical analyses

Statistical analyses were performed using SPSS version 28.0 (SPSS Inc., Chicago, IL, USA) To compare the two groups, Student's *t* test and Mann-Whitney *U* test were performed for parametric and non-parametric data, respectively. To analyze the negative geotaxis and active avoidance tests, two-way repeated measures analysis of variance (ANOVA) was used. Two-way ANOVA was used to analyze the rotarod treadmill test. Fisher's exact test was used to compare the proportion of fetal growth restriction (FGR) in the offspring. The sample size of each experiment was not determined using a power calculation. We established sample sizes of 6–7 dams in each group based on the preliminary experiments to determine the dose of L-NAME that would result in significant maternal hypertension and fetal growth restriction. Statistical significance was set at *p* < 0.05.

## 3. Results

### 3.1. PE rat model induced by L-NAME administration on GDs 15–20

**Figure 2:**
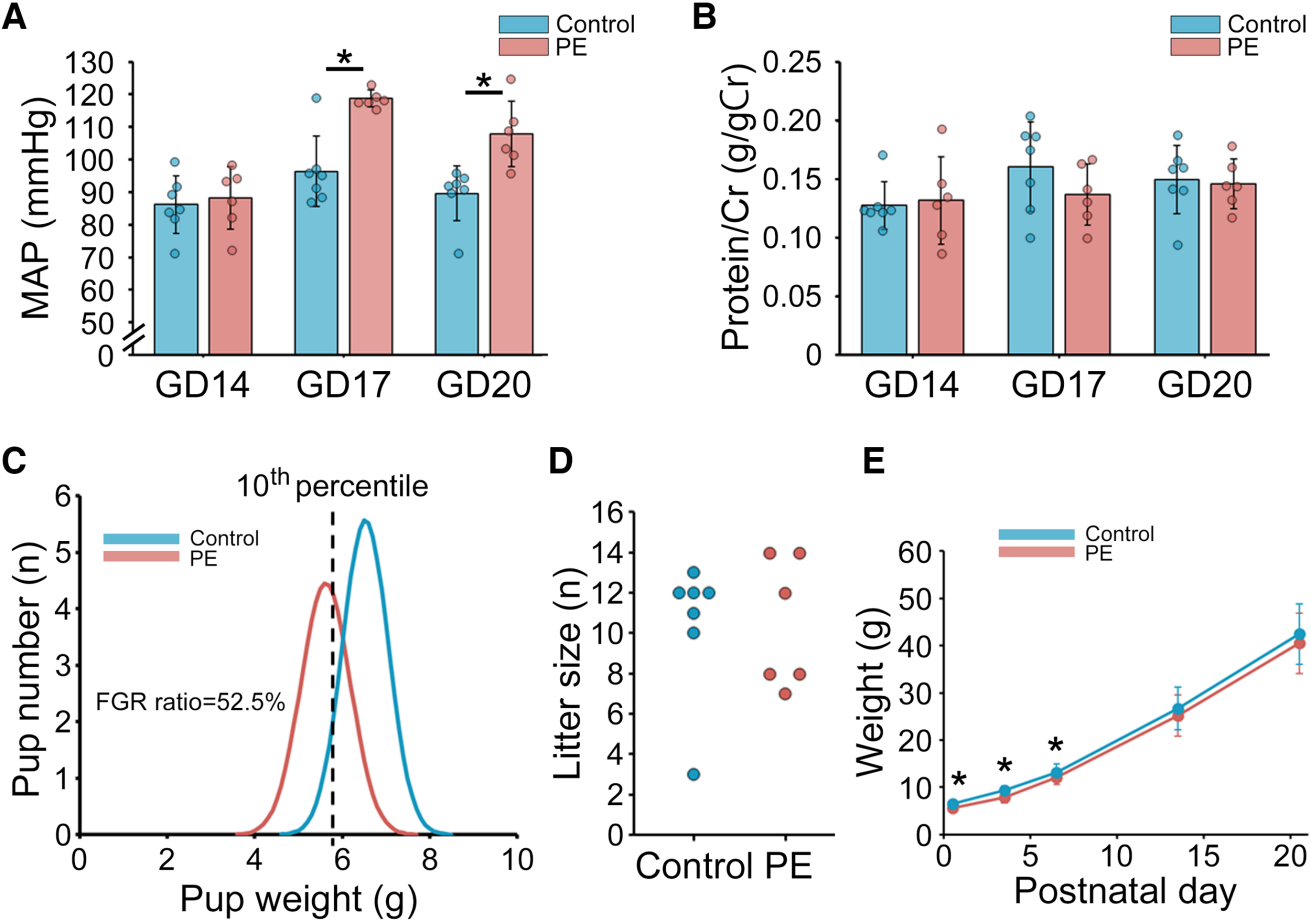
Maternal and offspring characteristics in the preeclampsia rat model induced by the administration of L-NAME. (**A**) MAP measured using the tail-cuff system on GDs 14, 17, and 20 (*n* = 6–7). (**B**) Urinary protein level on GDs 14, 17, and 20 (*n* = 6–7). Urinary protein level was evaluated with albumin/creatinine ratio (g/gCr). (**C**) Distribution of pup weight in the two groups. FGR was defined as birth weight falling below the 10th percentile based on the control group in this study (=5.76 g). (**D**) Litter size. (**E**) Postnatal offspring growth before weaning on postnatal day 21. Data are shown as the mean ± standard deviation. * indicates *p* < 0.05. PE, preeclampsia; MAP, mean arterial pressure; GD, gestational day; FGR, fetal growth restriction.

The mean arterial pressures on GDs 14, 17, and 20 were measured using the tail-cuff system (Figure 2A). The PE group showed a significantly higher mean arterial pressure than the control group (*p *< 0.01, GD17; *p* < 0.01, GD20). Urinary protein levels were evaluated using the albumin/creatinine ratio (g/g creatinine) (Figure 2B). The urinary protein levels in the PE group were comparable to those in the control group. We defined FGR as a birth weight below the 10th percentile based on the control group in this study (=5.76 g). Pup weight on PND 1 in the PE group was significantly lower and the FGR ratio (52.5%) was significantly higher than that in the control group (Figures 2C,E). There was no significant difference in the litter size between the two groups (Figure 2D). Postnatal growth in the PE group showed catch-up growth on PND 14 (Figure 2E).

### 3.2. Behavioral experiments of the PE model

**Figure 3:**
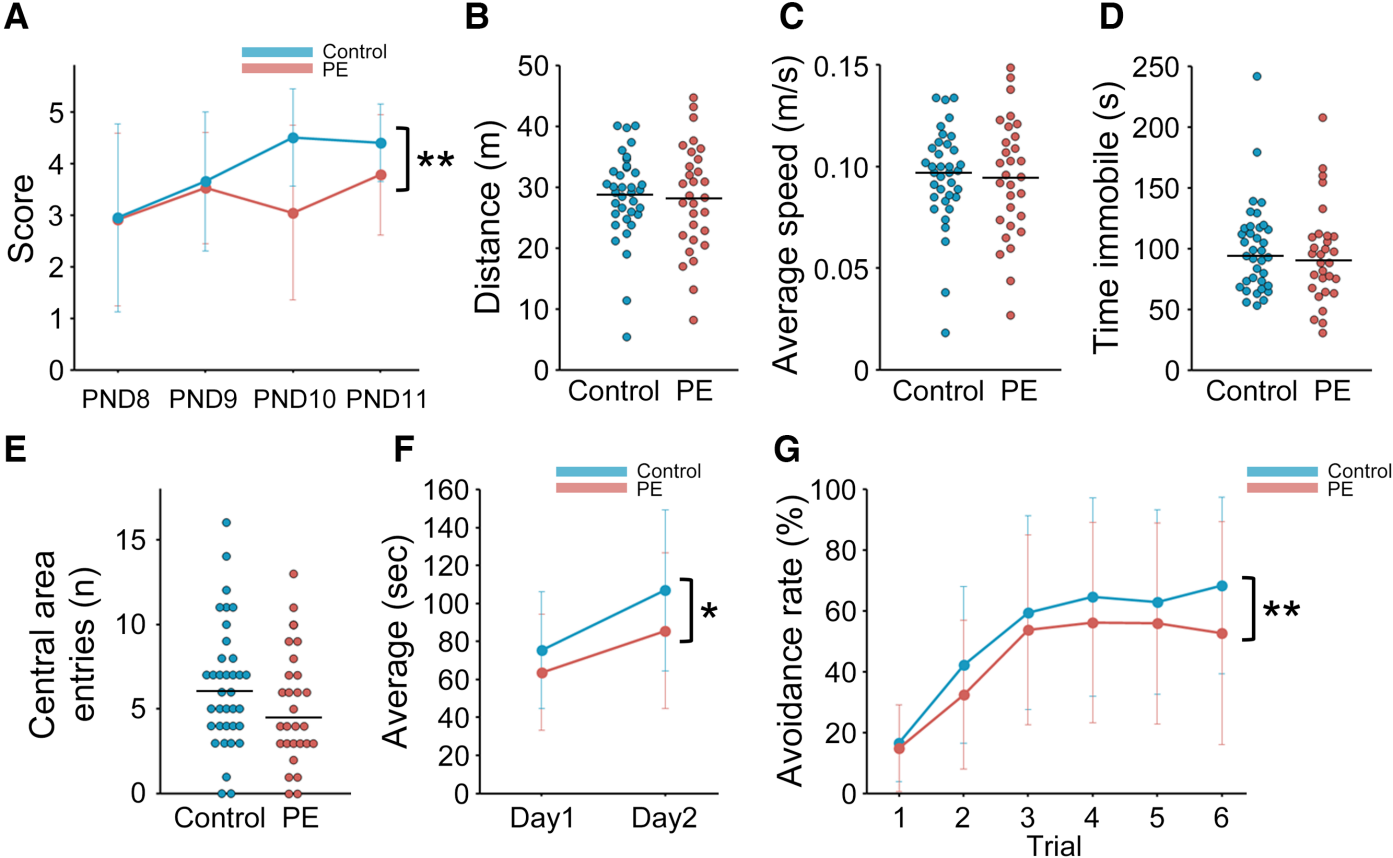
Behavioral experiments of the preeclampsia model. (**A**) Scores of negative geotaxis test on PNDs 8–11 (*n* = 20–23). (**B–E**) Open-field test on PND 35 (*n* = 30–36). Total distance moved (m), average speed (m/s), time immobile (s), and entries in central area (n) were evaluated by an automated tracking system. The median is indicated by the horizontal lines. (**F**) Rotarod treadmill test on PNDs 42–43 (*n* = 30–35). The average time on the rotating rod was evaluated. (**G**) Active avoidance test on PND 49 (*n* = 29–35). The trend of avoidance rates in six consecutive trials was presented. To analyze the negative geotaxis and active avoidance tests, two-way repeated measures ANOVA was used. To analyze the rotarod treadmill test, two-way ANOVA was used. Data are shown as the mean ± standard deviation. * and ** indicate *p* < 0.05 and *p* < 0.01, respectively. PE, preeclampsia; PND, postnatal day.

Male offspring in the L-NAME-induced PE group were evaluated for several neurological and neurodevelopmental features. Negative geotaxis, open-field, rotarod treadmill, and active avoidance tests were performed on PNDs 8–11, 35, 42–43, and 49, respectively (Figure 1). To evaluate neonatal reflexes, we performed a negative geotaxis test during the early postnatal period. Scores on PNDs 8 and 9 were comparable between the two groups; however, the scores of the PE group on PNDs 10 and 11 were lower than those of the control group (*p* < 0.01) (Figure 3A). For the open-field test, we evaluated the total distance traveled, average speed, time immobile, and central area entries. However, no significant differences were observed between the two groups (Figures 3B–E). The time spent on the rotating rod in the PE group was significantly shorter than that in the control group in the rotarod treadmill test (*p* = 0.05) (Figure 3F). The avoidance rate of the PE group was lower than that of the control group in the active avoidance test (*p* < 0.01) (Figure 3G).

### 3.3 NeuN immunostaining and positive cell counting in the hippocampus and cerebral cortex

**Figure 4:**
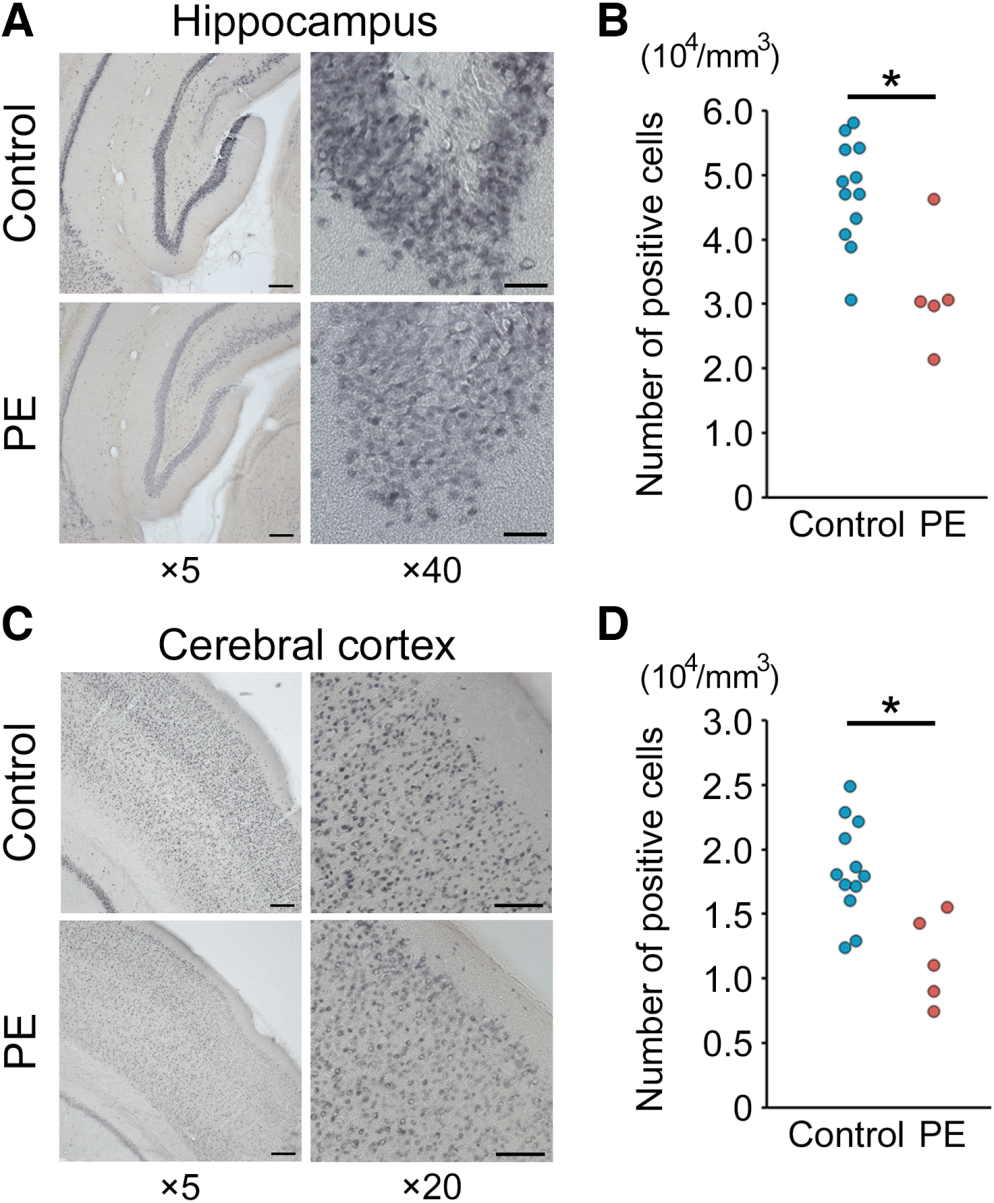
NeuN immunostaining and positive cell counting of the hippocampus and cerebral cortex. (**A**,**C**). Representative images (×5 and ×20–40) of NeuN staining of the hippocampal dentate gyrus and cerebral cortex on postnatal day 70. Scale bars = 200 μm (×5), 100 μm (×20), and 40 μm (×40). (**B,D**) Quantification of NeuN-positive cells (*n* = 5–12). Results are expressed as mean ± standard deviation. * indicates *p* < 0.05. PE, preeclampsia; NeuN, anti-neuronal nuclei.

Brain sections obtained on PND 70 were evaluated and compared between the two groups. Hematoxylin and eosin staining revealed no gross anatomical alterations in the PE group (data not shown). Figures 4A,C show representative images of NeuN immunostaining in the hippocampal dentate gyrus and cerebral cortex of the two groups. NeuN-positive cells in the PE group were significantly reduced in both the hippocampal dentate gyrus and cerebral cortex compared with those in the control group (*p* < 0.01 and *p* < 0.01, respectively) (Figures 4B,D).

### 3.4. Proteome analysis of CSF samples in male pups on PND 5

**Figure 5:**
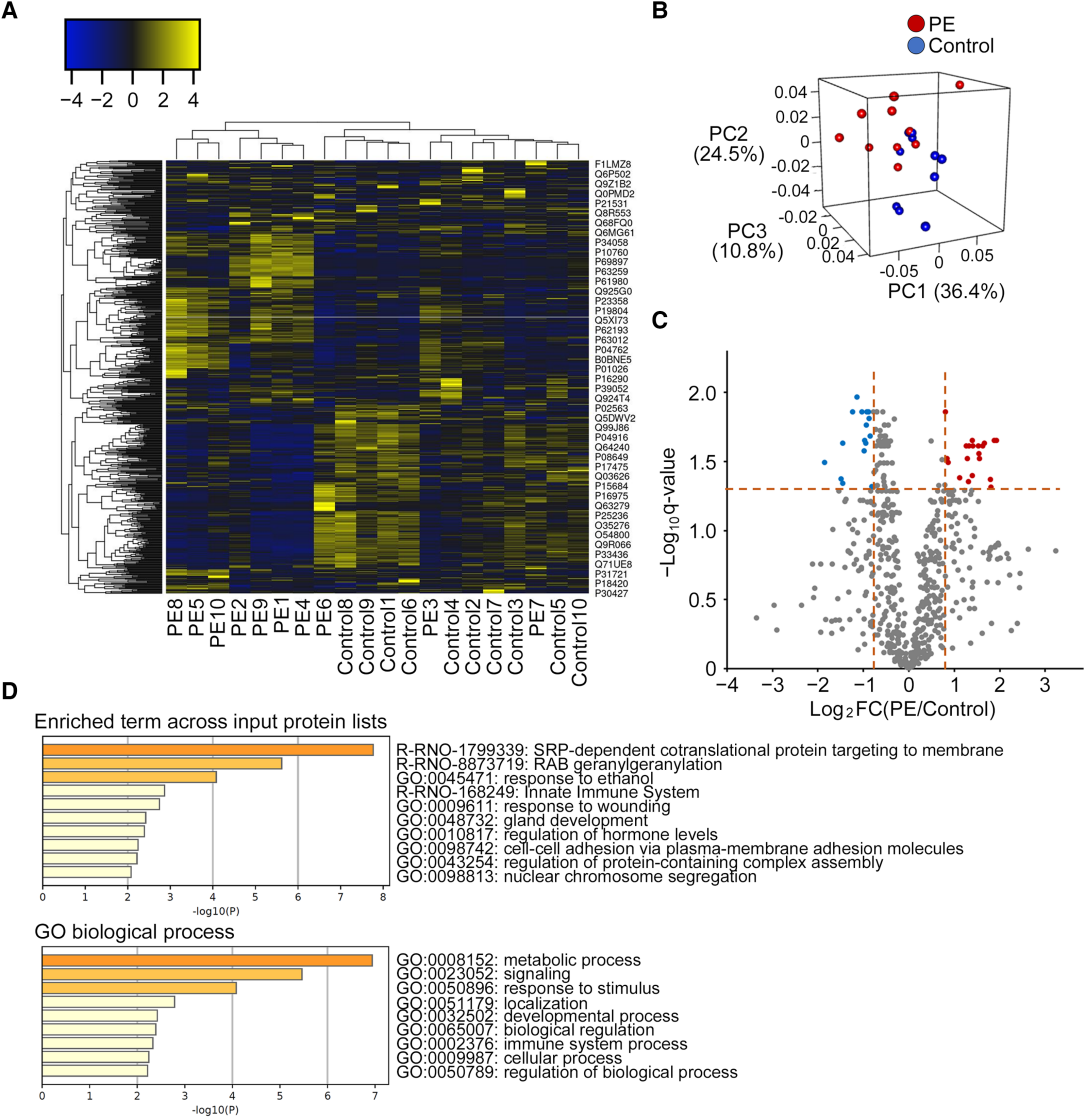
Distinct protein profiling of the cerebrospinal fluid in the preeclampsia and control groups. (**A**) Heat map with dendrograms of all identified protein expressions in the PE and control groups. Each column and row denotes a patient sample and specific protein, respectively. Dendrograms for samples and proteins are shown on the top and left of the heat map, respectively. (**B**) Principal component analysis shows the separation of samples from the PE and control groups. (**C**) Differentially expressed proteins shown by volcano plot (fold change vs. *q*-value). Red dots represent upregulated proteins (*n* = 20), blue dots represent downregulated proteins (*n* = 16), and gray dots represent no differentially expressed proteins (*n* = 586). (**D**). Gene ontology analyses of differentially expressed proteins (*n* = 32) by Metascape (top enriched term and gene ontology biological process) after filtering the rat database. PE, preeclampsia; FC, fold change; PC, principal component; GO, gene ontology.

Among the 1270 proteins detected by LC-MS/MS, 622 proteins were present in at least four samples among the 10 samples in each group. A heat map with dendrograms of the samples and identified proteins is presented in Figure 5A. PCA showed that most samples achieved a clear separation between the PE and control groups in three dimensions (Figure 5B). A volcano plot was created to visualize the differentially expressed proteins (Figure 5C). A total of 36 proteins were matched for the criteria (fold change and *q*-value) of differentially expressed proteins, and 32 proteins were identified as differentially expressed proteins after filtering the rat database (Supplementary Table S1). Among the 32 proteins, 18 were upregulated and 14 were downregulated in the CSF of the PE group compared with that of the control group. The statistically enriched terms across the input protein lists and top-level GO biological processes are presented in Figure 5D. Among these 32 differentially expressed proteins, Rab35, Rab3a, Tcp1, Gstm1, Rack1, Il1rap, Ccdc60, Esam, and Psmb6 are closely associated with neurological disorders including ASD, schizophrenia, and Alzheimer's disease. In addition, some ribosomal proteins, including Rps15a, Rpl18, Rpl15, Rps3a, Rpl22, and Rpl6, were significantly increased in the PE group.

## 4. Discussion

In this study, we sought to investigate the mechanism underlying the increased risk of subsequent neurodevelopmental and neuropsychiatric disorders in offspring attributed to intrauterine exposure to maternal PE. We initially evaluated whether the offspring of L-NAME-induced PE model rats manifested several neurological features similar to those in humans, and then we evaluated the alterations in protein profiling of CSF in the PE model. The main findings of this study were as follows: First, offspring in the L-NAME-induced PE model showed FGR, postnatal catch-up growth, and neurological abnormalities (e.g., delayed neurodevelopment in the early postnatal period [negative geotaxis], delayed motor coordination and learning skills [rotarod treadmill], and poorer memory skills [active avoidance test] in the juvenile period), consistent with the clinical manifestations of children born to mothers with PE. Second, significant pathological changes (e.g., a decrease in neuronal cells in the hippocampus and cortex) and altered protein profiling of the CSF were observed. Third, unique protein signatures related to ER translocation, Rab proteins, and ribosomal proteins were identified by proteome analysis of CSF. Our study indicated that these alterations may cause adverse neurological consequences later in life.

Several previous studies demonstrated that the offspring of the L-NAME-induced PE model rats showed various abnormal neuropathological findings including neurotoxicity, upregulated expression of hippocampal glucocorticoid receptors, disrupted neurogenesis, and acute neuronal damage (27–30); however, little is known regarding whether this L-NAME-induced PE model indeed shows changes in anxiety, cognition, and memory-related behaviors. In this study, we found abnormalities in the negative geotaxis, rotarod treadmill, and active avoidance tests, indicating that the offspring of the PE model showed impaired neurodevelopment in innate postural response, balance and motor coordination, and fear-motivated associative learning and memory. These results are consistent with two previous studies that demonstrated impaired spatial learning and memory using the water maze test in an L-NAME model (28, 29). In addition to the L-NAME model, the offspring of the PE rat model induced by drinking water with 1.8% sodium chloride during pregnancy remained on the rotating rod for a significantly shorter period than control rats (31). However, careful attention is necessary to the numerous variations of the PE phenotypes in each L-NAME-induced PE model. In contrast, the offspring of another PE mouse model induced by arginine vasopressin showed no difference in the latency of fall from the rod (6). In agreement with our study, offspring in the arginine vasopressin PE mouse model showed no adverse effects on open-field behavior (6). The negative geotaxis test is an early behavioral test to evaluate motor development and sensory, vestibular, and proprioceptive functions and is useful and validated in the setting of various neurological and neurodevelopmental disorders (e.g., ASD and cerebral palsy) (18, 32).

To date, two studies have investigated the CSF profiling of mothers with PE; however, to the best of our knowledge, no studies have demonstrated alterations in CSF profiles in the offspring. Ciampa et al. demonstrated that differentially expressed proteins in the CSF of PE mothers converge to four signaling molecules, including TGF-β, vascular endothelial growth factor A, angiotensinogen, and IL-6, suggesting that neurological maternal complications of PE, such as eclampsia and posterior reversible encephalopathy syndrome, are associated with vascular remodeling, inflammation, neuronal growth, signaling, and electrophysiology (33). Güzel et al. also demonstrated that some differentially expressed proteins were calcium-binding proteins, suggesting a close association between PE and calcium-binding proteins (34). As with these two studies on maternal CSF, investigation of CSF protein profiling in the offspring of the L-NAME-induced PE model may facilitate the elucidation of the mechanisms underlying subsequent neurological disorders (35). In our study, heat map and PCA analyses of the CSF protein profiles revealed significantly different protein signatures between the PE and control groups. Among the 32 differentially expressed proteins, Rab35, Rab3a, Tcp1, Gstm1, Rack1, Il1rap (ASD) (36–40), Ccdc60, Esam (schizophrenia) (41, 42), and Psmb6 (Alzheimer's disease) (43) are associated with various nervous system disorders. The RAB family genes have been identified as ASD-associated genes (36, 44). Rab35 is involved in synaptic vesicle transport, and synaptic disturbance is recognized as a molecular disruption in various neurodevelopmental disorders, including ASD (36, 45). In addition, we found significantly increased levels of some ribosomal proteins in the CSF of the rats in the PE group. Ribosomes are essential for the growth, development, and function of all organisms and play a central role in protein synthesis (46). Ribosomes are found in large numbers within cells, and are composed of ribosomal RNA molecules and associated ribosomal proteins that form a ribonucleoprotein complex that drives the translation process. Altered ribosomal protein synthesis can lead to impaired neuronal function, disrupted synaptic connectivity, and accumulation of abnormal protein aggregates in neurological conditions (47, 48). Growing evidence has demonstrated that dysfunction of ribosomes can be linked to a range of neurodegenerative disorders such as Alzheimer's disease, Parkinson's disease, and amyotrophic lateral sclerosis (48–50). Therefore, exposure to PE *in utero* may increase the incidence of neuropsychiatric disorders by altering ribosomal protein synthesis in the nervous system.

Regarding the GO analyses based on these 32 differentially expressed proteins, signal recognition particle (SRP)-dependent cotranslational proteins targeting the membrane and RAB geranylgeranylation were listed in the top two pathways. The SRP-dependent cotranslational protein targeting the membrane is associated with the cotranslational delivery of integral membrane proteins for translocation into the endoplasmic reticulum (ER) by an SRP (51). The inhibition of ER translocation promotes ER stress (52), which enhances neuronal differentiation and is related to ASD pathogenesis (53). Rab proteins, members of the Ras superfamily, constitute the largest family of small GTPases. Rab GTPases function as molecular switches that control membrane trafficking to regulate the formation, transport, tethering, and fusion of transport vesicles in various biological processes, including neurological functions (e.g., synaptic function, neurite growth and remodeling, and general nervous system development) (54). Rab GTPase dysfunction including impaired RAB geranylgeranylation (i.e., essential post-translational modification that allows Rab proteins to interact with intracellular membranes) is associated with the pathogenesis of neurodegeneration (e.g., Alzheimer's and Parkinson's diseases) and neurogenesis impairment (e.g., ASD) (55–59). These unique protein signatures related to ER translocation and Rab proteins in the PE model may be associated with subsequent neurodevelopmental disorders in the offspring (54).

The first strength of this study is that we evaluated the protein profile of offspring CSF for the first time. In addition, we obtained the CSF at an early stage of life (PND 5). Although CSF collection on PND 5 requires practice and skills, it has been unaffected by various postnatal factors (e.g., diet, dam, and various environments) and may be better for evaluating CSF protein profiling compared with that obtained in adults. Second, GO analyses led to a new hypothesis that dysfunction of ER translocation and Rab GTPase may contribute to subsequent neurodevelopmental disorders in offspring. Finally, although little is known about the neurobehavioral abnormalities of offspring in the L-NAME-induced PE model, except for the water maze test, we performed several behavioral tests to evaluate neurodevelopment.

This study has several limitations. First, we could not validate the proteome analyses because the sample volume of the CSF was quite small, and the protein concentration in the CSF was considerably low (approximately 1/200) compared with that of serum samples (60). Further study is required to identify candidate proteins that may disrupt neurodevelopment and to perform functional analyses. Second, there are many variations of the L-NAME-induced PE model with respect to L-NAME dose, route of administration, and duration of administration during gestation. Our PE model did not perfectly exhibit all the features of PE, including urinary protein levels which is a key feature of PE. Therefore, some individuals may consider it a model of gestational hypertension rather than a PE model. Although numerous PE animal models have been established, such as the adenovirus sFlt-1 model, angiotensin II-induced model, and reduced uterine perfusion pressure model, none perfectly mimic all the key features of PE (61). Finally, we evaluated the neurological characteristics of only male, not female, offspring, to omit the sex-differential effect and the effect of the estrous cycle on behavioral experiments (62, 63). Further studies are warranted to include female offspring to evaluate the sex-differential effect.

In conclusion, we demonstrated that the offspring in the L-NAME-induced PE model exhibited key features of neurodevelopmental abnormalities, such as delayed innate postural response near birth, delayed motor coordination and learning skills, and fear-motivated associated learning and memory deficit, but not the features of anxiety in early adolescence. This L-NAME model would be useful for the evaluation of offspring neurodevelopmental consequences. In addition, we observed altered CSF protein profiles in the PE model, suggesting that the unique protein signatures related to ER translocation, Rab proteins, and ribosomal proteins may be associated with subsequent adverse neurodevelopment in the offspring. Further research is necessary to discover and validate candidate proteins that may disrupt subsequent neurodevelopment.

## Data Availability

The datasets presented in this study can be found in online repositories. The names of the repository/repositories and accession number(s) can be found below: https://repository.jpostdb.org/, JPST002007.
