# Supplementary material for: Altered offspring neurodevelopment in an L-NAME-induced preeclampsia rat model
Source: Front Pediatr. 2023 Jul 13;11:1168173. doi: 10.3389/fped.2023.1168173 (PMC10373593; doi:10.3389/fped.2023.1168173)
Supplement: Supplementary file 1 [file Table1.docx]

Supplementary Material

Altered offspring neurodevelopment in an L-NAME-induced preeclampsia rat model

Noriyuki Nakamura^1,2^, Takafumi Ushida^1,3,^*, Atsuto Onoda^4^, Kazuto Ueda^5^, Ryosuke Miura^5^, Toshihiko Suzuki^5^, Satoru Katsuki^1^, Hidesuke Mizutani^1^, Kosuke Yoshida^1^, Sho Tano^1^, Yukako Iitani^1^, Kenji Imai^1^, Masahiro Hayakawa^5^, Hiroaki Kajiyama^1^, Yoshiaki Sato^5,^*, Tomomi Kotani^1,3^

*** Correspondence:** Takafumi Ushida: [u-taka23@med.nagoya-u.ac.jp](mailto:u-taka23@med.nagoya-u.ac.jp); Yoshiaki Sato: yoshiaki@med.nagoya-u.ac.jp

# Supplementary Table

Supplementary Table 1. Identified and mapped differentially expressed proteins between the two groups.

| Accession number | Gene ID | Gene Symbol | Description | *q* value | log2FC | Sample number in PE and control |
| --- | --- | --- | --- | --- | --- | --- |
| Increase |  |  |  |  |  |  |
| P62246 | 117053 | Rps15a | ribosomal protein S15a | 0.022 | 1.935 | 9/10 and 4/10 |
| Q5U316 | 288700 | Rab35 | RAB35, member RAS oncogene family | 0.022 | 1.880 | 9/10 and 4/10 |
| P12001 | 81766 | Rpl18 | ribosomal protein L18 | 0.049 | 1.806 | 9/10 and 4/10 |
| P61314 | 245981 | Rpl15 | ribosomal protein L15 | 0.043 | 1.796 | 9/10 and 6/10 |
| P63012 | 25531 | Rab3a | RAB3A, member RAS oncogene family | 0.023 | 1.667 | 9/10 and 4/10 |
| P62824 | 171058 | Rab3c | RAB3C, member RAS oncogene family | 0.024 | 1.634 | 9/10 and 4/10 |
| P28480 | 24818 | Tcp1 | t-complex 1 | 0.030 | 1.552 | 9/10 and 6/10 |
| Q6NYB7 | 81754 | Rab1a | RAB1A, member RAS oncogene family | 0.024 | 1.538 | 9/10 and 5/10 |
| P04905 | 24423 | Gstm1 | glutathione S-transferase mu 1 | 0.024 | 1.414 | 9/10 and 6/10 |
| Q5M7U6 | 289820 | Actr2 | actin related protein 2 | 0.040 | 1.398 | 9/10 and 4/10 |
| P49242 | 29288 | Rps3a | ribosomal protein S3a | 0.022 | 1.396 | 9/10 and 9/10 |
| Q66HR2 | 114764 | Mapre1 | microtubule-associated protein, RP/EB family, member 1 | 0.024 | 1.322 | 9/10 and 4/10 |
| P11348 | 64192 | Qdpr | quinoid dihydropteridine reductase | 0.044 | 1.321 | 8/10 and 5/10 |
| P47198 | 81768 | Rpl22 | ribosomal protein L22 | 0.030 | 1.287 | 9/10 and 7/10 |
| P63245 | 83427 | Rack1 | receptor for activated C kinase 1 | 0.024 | 1.266 | 10/10 and 10/10 |
| P21533 | 117042 | Rpl6 | ribosomal protein L6 | 0.042 | 1.117 | 10/10 and 10/10 |
| Q08163 | 64185 | Cap1 | cyclase associated actin cytoskeleton regulatory protein 1 | 0.030 | 0.844 | 10/10 and 9/10 |
| P28073 | 29666 | Psmb6 | proteasome 20S subunit beta 6 | 0.014 | 0.811 | 10/10 and 9/10 |
| Decrease |  |  |  |  |  |  |
| P26051 | 25406 | Cd44 | CD44 molecule (Indian blood group) | 0.049 | −0.814 | 10/10 and 10/10 |
| Q5DWV2 | 29162 | Cdh7 | cadherin 7 | 0.021 | −0.840 | 9/10 and 10/10 |
| Q3ZAV0 | 498190 | Ccdc60 | coiled-coil domain containing 60 | 0.016 | −0.868 | 9/10 and 10/10 |
| Q63621 | 25466 | Il1rap | interleukin 1 receptor accessory protein | 0.014 | −0.879 | 10/10 and 10/10 |
| P14925 | 25508 | Pam | peptidylglycine alpha-amidating monooxygenase | 0.017 | −0.929 | 10/10 and 10/10 |
| O55004 | 56759 | Rnase4 | ribonuclease A family member 4 | 0.023 | −0.944 | 10/10 and 10/10 |
| Q9EQV8 | 365466 | Cpn1 | carboxypeptidase N subunit 1 | 0.022 | −0.963 | 10/10 and 10/10 |
| P01346 | 24483 | Igf2 | insulin-like growth factor 2 | 0.027 | −0.983 | 10/10 and 10/10 |
| Q8R2H5 | 291132 | Gpld1 | glycosylphosphatidylinositol specific phospholipase D1 | 0.014 | −1.029 | 10/10 and 10/10 |
| P27657 | 25702 | Pnlip | pancreatic lipase | 0.014 | −1.231 | 10/10 and 10/10 |
| Q9ERQ6 | 50568 | Cspg5 | chondroitin sulfate proteoglycan 5 | 0.046 | −1.454 | 4/10 and 10/10 |
| Q6AYD4 | 300519 | Esam | endothelial cell adhesion molecule | 0.023 | −1.454 | 8/10 and 10/10 |
| Q6L711 | 292126 | Habp2 | hyaluronan binding protein 2 | 0.042 | −1.488 | 7/10 and 10/10 |
| D3ZF92 | 316256 | Tnfrsf21 | TNF receptor superfamily member 21 | 0.032 | −1.844 | 5/10 and 10/10 |

FC, fold change; PE, preeclampsia.
